# Deep-learning-based pelvic automatic segmentation in pelvic fractures

**DOI:** 10.1038/s41598-024-63093-w

**Published:** 2024-05-28

**Authors:** Jung Min Lee, Jun Young Park, Young Jae Kim, Kwang Gi Kim

**Affiliations:** 1 Department of Computer Engineering, College of IT Convergence, Gachon University, Seongnam, Republic of Korea; 2 Department of Biomedical Engineering, College of Medicine, Gachon University, Incheon, Republic of Korea; 3 Medical Device R&D Center, Gachon University Gil Hospital, Incheon, Republic of Korea; 4 Department of Health Sciences and Technology, Gachon Advanced Institute for Health Sciences and Technology, Gachon University, Incheon, Republic of Korea

**Keywords:** Pelvic fracture, Segmentation, Deep learning, Biomedical engineering, Diagnostic markers, Diagnosis

## Abstract

With the recent increase in traffic accidents, pelvic fractures are increasing, second only to skull fractures, in terms of mortality and risk of complications. Research is actively being conducted on the treatment of intra-abdominal bleeding, the primary cause of death related to pelvic fractures. Considerable preliminary research has also been performed on segmenting tumors and organs. However, studies on clinically useful algorithms for bone and pelvic segmentation, based on developed models, are limited. In this study, we explored the potential of deep-learning models presented in previous studies to accurately segment pelvic regions in X-ray images. Data were collected from X-ray images of 940 patients aged 18 or older at Gachon University Gil Hospital from January 2015 to December 2022. To segment the pelvis, Attention U-Net, Swin U-Net, and U-Net were trained, thereby comparing and analyzing the results using five-fold cross-validation. The Swin U-Net model displayed relatively high performance compared to Attention U-Net and U-Net models, achieving an average sensitivity, specificity, accuracy, and dice similarity coefficient of 96.77%, of 98.50%, 98.03%, and 96.32%, respectively.

## Introduction

Pelvic fractures, second only to skull fractures in terms of mortality and complication risk, are becoming more frequent because of the recent increase in traffic accidents^1^. Interest in pelvic fractures has increased since the 1960s, primarily due to intra-abdominal hemorrhage, the leading cause of death related to pelvic fractures. Since the 1970s, advancements in computed tomography (CT) imaging, external fixation, and pelvic packing have significantly improved the management, diagnosis, and treatment of pelvic fractures^2^. However, an accurate diagnosis of pelvic fractures remains challenging owing to the complex three-dimensional (3D) nature of the pelvic structure, making it difficult to assess using conventional radiographic techniques^3^. Simple radiographic examinations may suggest the possibility of a fracture, whereas attempting to confirm it through CT often results in either no fracture or overestimation of the fracture extent^4^. This indicates the limitations of conventional radiography in precisely assessing the presence or extent of fractures^5^.

Recent research on medical imaging has demonstrated the potential for various segmentation tasks, such as bone^6^, pulmonary vessels ^7^, and pelvic and sacral bones ^8^. Wang et al.^9^ attempted to segment the pelvis using a deep learning method and achieved a relatively high detection performance, with an accuracy of 91% and a sensitivity of 84%. Liu et al.^10^ used a 3D U-Net model to segment the pelvis, demonstrating high accuracy with a dice similarity coefficient (DSC) of 84%. Yuan conducted another study on pelvic segmentation using 11 models and verified their performances. The dataset comprised 14,487 images obtained from 42 patients. In three experiments, BiseNet and LedNet achieved accuracies of 99.49% and 99.41%, respectively^11^.

Recently, several studies have been performed to detect organ/bone fragmentation and lung nodules. Wu et al.^12^ used Attention U-Net to attempt organ segmentation in abdominal CT images and achieved high accuracy. Noguchi et al.^13^ performed bone segmentation on whole-body CT images using a U-Net based architecture. Recently, Wasserthal et al.^14^ automatically segmented all anatomical structures identifiable in the CT images of each body using no-new-U-Net (nnU-Net).

Considerable research has been conducted on deep-learning models to diagnose lesions and fractures. Kai et al.^15^ utilized the Swin U-Net for tumor segmentation in breast cancer to enable rapid diagnosis. Gao et al.^16^ proposed a lightweight Swin U-Net model for segmenting COVID-19 lesions in CT images, achieving excellent segmentation results in multiple lesion regions. Urakawa et al.^17^ used the VGG model to classify pelvic fractures, focusing on straightforward lesion classification.

Although numerous attempts have been made to segment tumors and organs, studies aiming to develop clinically useful algorithms for bone and pelvis segmentation using these models are limited.

In this study, we examined previous research to explore the potential of deep learning to achieve accurate pelvic region segmentation in X-ray images. When diagnosing pelvic fractures on radiographs, the extent of the fracture in the pubic bone, ilium, or ischium is crucial. Most fractures can be detected on radiographs; however, rapid diagnosis is challenging, particularly for fractures in the pubic and ischial bones or fragmented bone pieces^18^. Therefore, in this study, the pelvic bone region in X-ray images was segmented using Attention U-Net, Swin U-Net, and U-Net in normal patients as well as those with fractures. In patients with severe fractures, medical experts may not be able to identify all the broken bones. In this case, the deep learning model can segment the fine bone fragments. Even in patients with minor fractures, a fracture diagnosis can be quickly made by first finding and showing the pelvic bone area to a medical professional. To improve the segmentation performance of the pelvic bone region in X-ray images, preprocessing was followed by the adjustment of deep learning model hyperparameter.

## Methods

### Research environment

The experiments in this study used a system comprising an NVIDIA TESLA P40 graphics processing unit (NVIDIA, Santa Clara, CA, USA), an Intel Xeon E5-2630 v4 CPU (Intel, Santa Clara, CA, USA), 32 GB of RAM, and the Ubuntu 20.04.6 LTS operating system. The programming language used for the experiment was Python (version 3.7.16). The libraries used for preprocessing and training the deep learning models were TensorFlow software (version. 2.6.0), Keras (version. 2.6.0) for various functions supporting deep learning model design and training, unified device architecture (version. 11.2.0) for massive computational processing as a graphics processing device development tool, open computer vision (version. 4.6.0.66) providing various functions for image processing, and matplotlib (version. 3.5.2) for data visualization.

### Data

**Figure 1:**
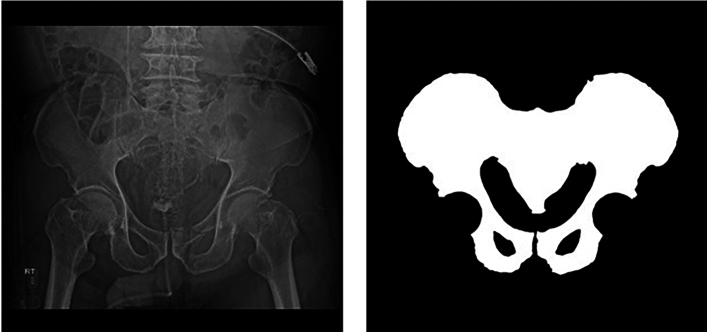
Pelvic ring segmentation.

The X-ray images were collected from Gachon University Gil Hospital between January 2015 and December 2022. The dataset comprised X-ray images from 773 adults aged 18 and above diagnosed with pelvic fractures and 167 individuals without pelvic fractures. This study received approval from the Gachon University Gil Hospital Clinical Research Ethics Review Committee, and the need for informed consent was waived due to the retrospective nature of the study (GAIRB2022-153). All experimental protocols were performed in accordance with the relevant guidelines and regulations of the Declaration of Helsinki. The location of the pelvic region was determined by referencing the pelvic AP radiographic findings of radiologists and CT scans. Two trauma surgeons with more than ten years of experience confirmed all fracture sites on the pelvic AP X-ray radiographs. Subsequently, regions of interest (ROIs) were delineated along the boundaries of the pelvic ring for pelvic segmentation (Fig. 1). The ROIs in polygon form were determined using ImageJ software (version. 1.53t, National Institutes of Health, Bethesda, MD, USA).

### Data preprocessing

The variations in the imaging environment of some collected pelvic AP radiographs pose difficulties in adequately identifying the pelvic region. Such images can potentially hinder the effective training of deep-learning algorithms. Thus, data preprocessing becomes a fundamental task for automated pelvic bone segmentation, serving as a crucial step in enhancing the learning capabilities and predictive performance of the model while extracting pertinent information from the data.

Therefore, before the analysis, a histogram equalization preprocessing step was applied to all the image data. In addition, efficient training of convolutional neural networks in deep learning requires consistent dimensions of the input and output images. Hence, in this study, a preprocessing step involving zero padding of the original images was performed to ensure uniform dimensions in terms of both width and height. Subsequently, the images were resized to a uniform size of 512 pixels, with both width and height set to 512 pixels.

### Convolutional neural network model for deep learning

**Figure 2:**
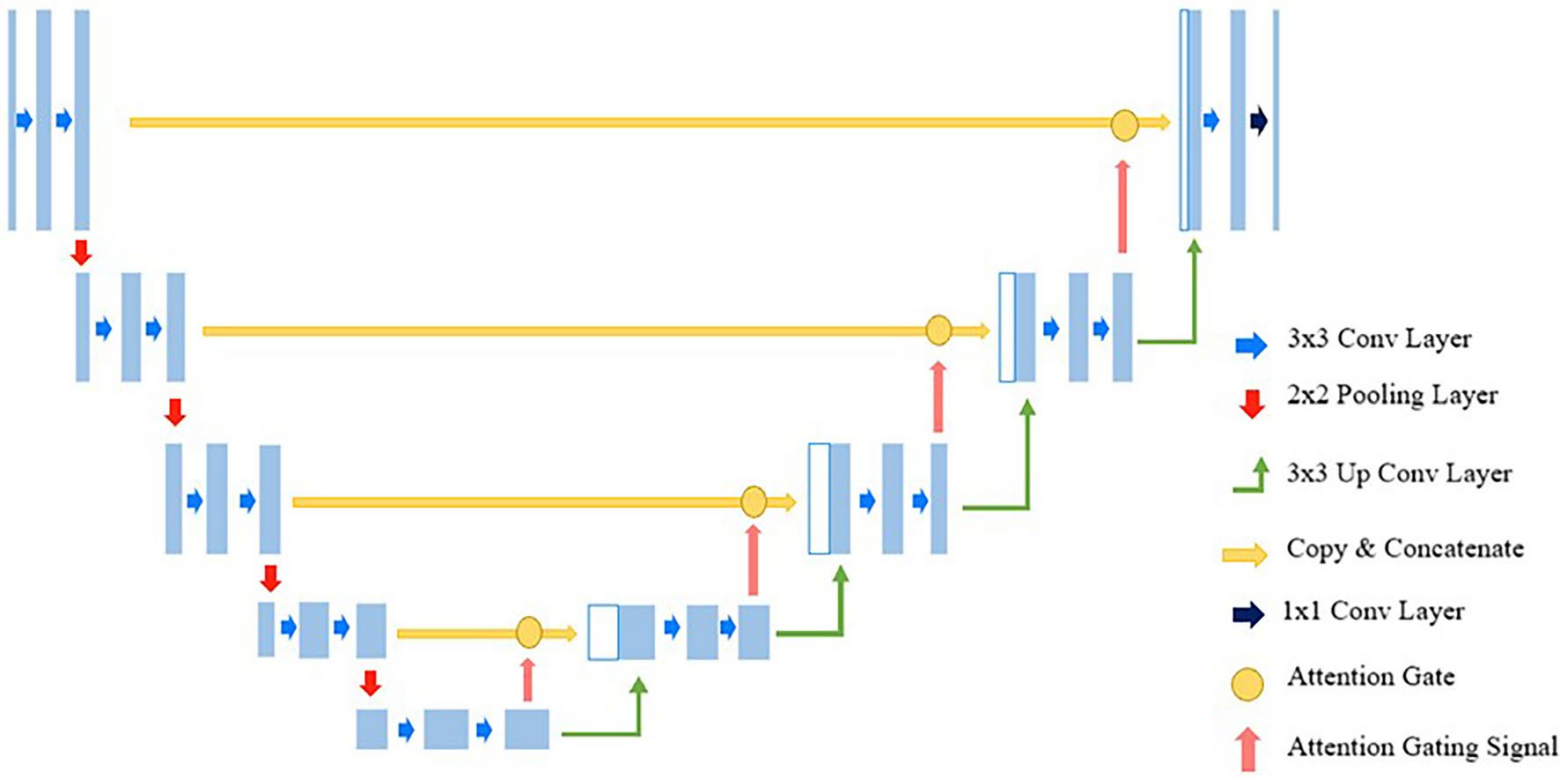
Architecture of Attention U-Net model.

**Figure 3:**
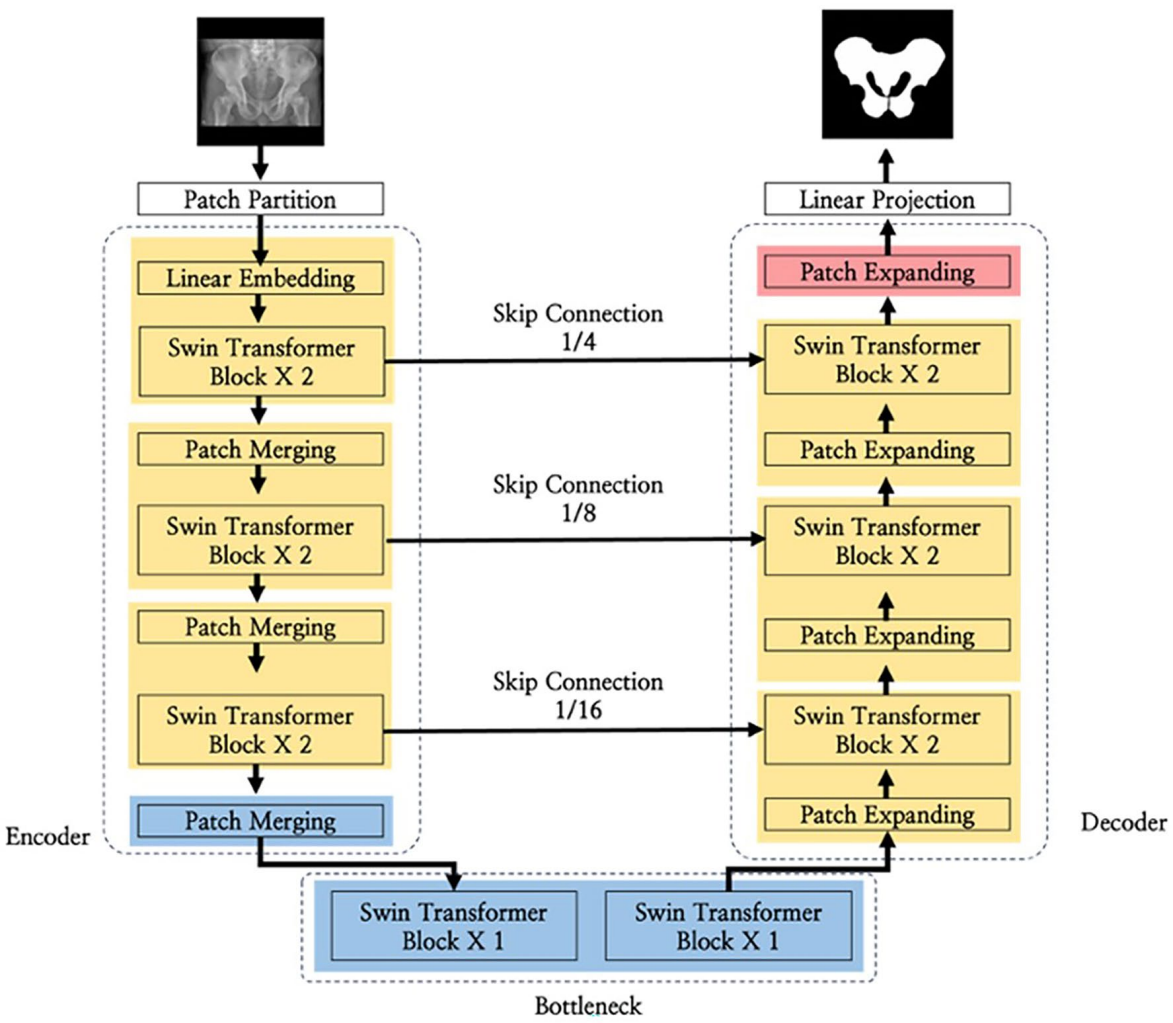
Architecture of Swin U-Net model.

**Figure 4:**
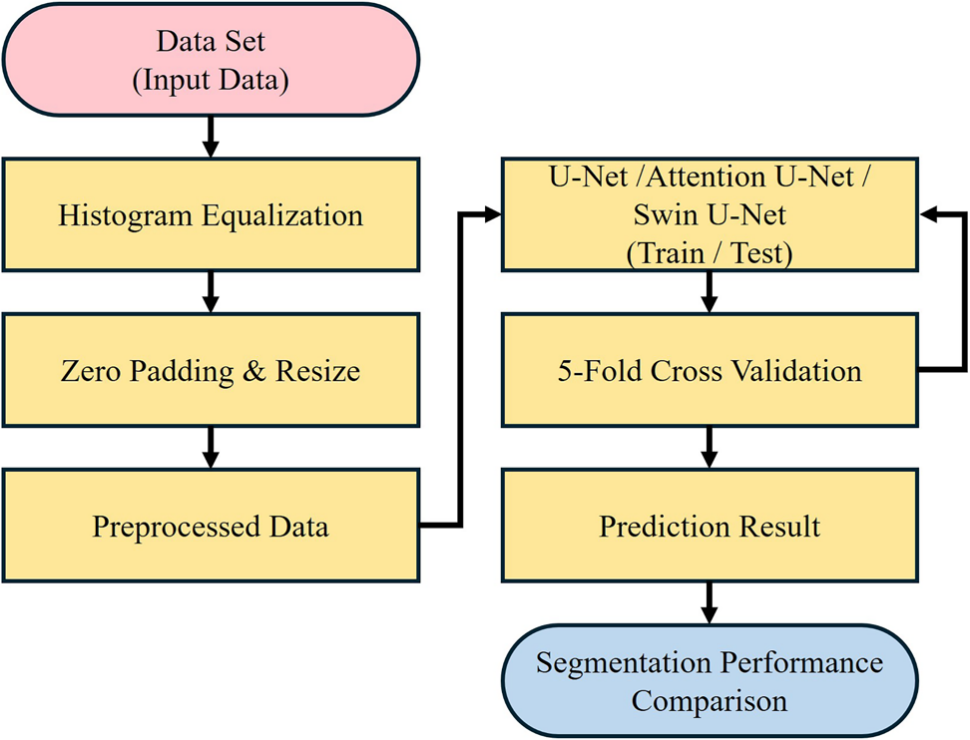
Flowchart of the proposed method.

In this study, the Attention U-Net, Swin U-Net, and U-Net models were employed for pelvic segmentation training. The Attention U-Net model is an adaptation of the U-Net architecture that integrates attention gates, as shown in Fig. 2. Despite sharing a similar architecture to that of U-Net, it incorporates attention gates in the decoder section to adjust the weights of regions that are not of interest. These aspects are distinguished by omitting regions deemed inconsequential or irrelevant to the prediction or classification outcomes. This process enhances the emphasis on critical areas, leading to more precise segmentation results. The Swin U-Net model combines Swin Transformer blocks with the U-Net architecture, as shown in Fig. 3. Swin transformers have demonstrated excellent performances in image classification and visual tasks, employing the self-attention structure of transformers to model the relationships between pixels, thereby enabling the capture of global contextual information. Moreover, the design includes an encoder-decoder structure specialized for medical image segmentation tasks. For optimization, the Adam optimizer was utilized with a batch size of one and learning rate of 0.001, training for 100 epochs, with model weights updated in each epoch. Figure 4 shows the flowchart proposed in this study. An early stopping function was incorporated to terminate the learning process early and prevent overfitting. When the validation loss did not improve, the patience was set to 30 for training. In addition, to continuously observe validation loss and dynamically adjust the learning rate, the ReduceLROnPlateau function was added to modify the model learning by reducing the learning rate when no change was observed in a certain epoch. To evaluate and compare the performance of both models, we divided the dataset into five segments and conducted a five-fold cross-validation. By comparing the visual analysis results of the medical experts with the prediction results of the deep learning model, the true positive (TP), false negative (FN), true negative (TN), and false positive (FP) were obtained. We assessed and compared the pelvic segmentation performances of the models using sensitivity, specificity, accuracy, and DSC as metrics, defined as follows: sensitivity: TP/(TP + FP); specificity: TN/(TN + FP); and accuracy: (TP + TN)/(TP + TN + FP + FN). Sensitivity measures the effectiveness of the model in detecting the actual pelvic region in ROI images. Specificity measures how well the model detects non-pelvic areas and indicates the ratio of correctly predicted areas outside the pelvic region. Accuracy measures the ratio of pixels predicted as the pelvis among all pixels, whereas DSC measures the similarity between the predicted and actual pelvic regions in the ROI images^19^. Higher values for each metric indicate better performance in pelvic region segmentation.

## Results

**Figure 5:**
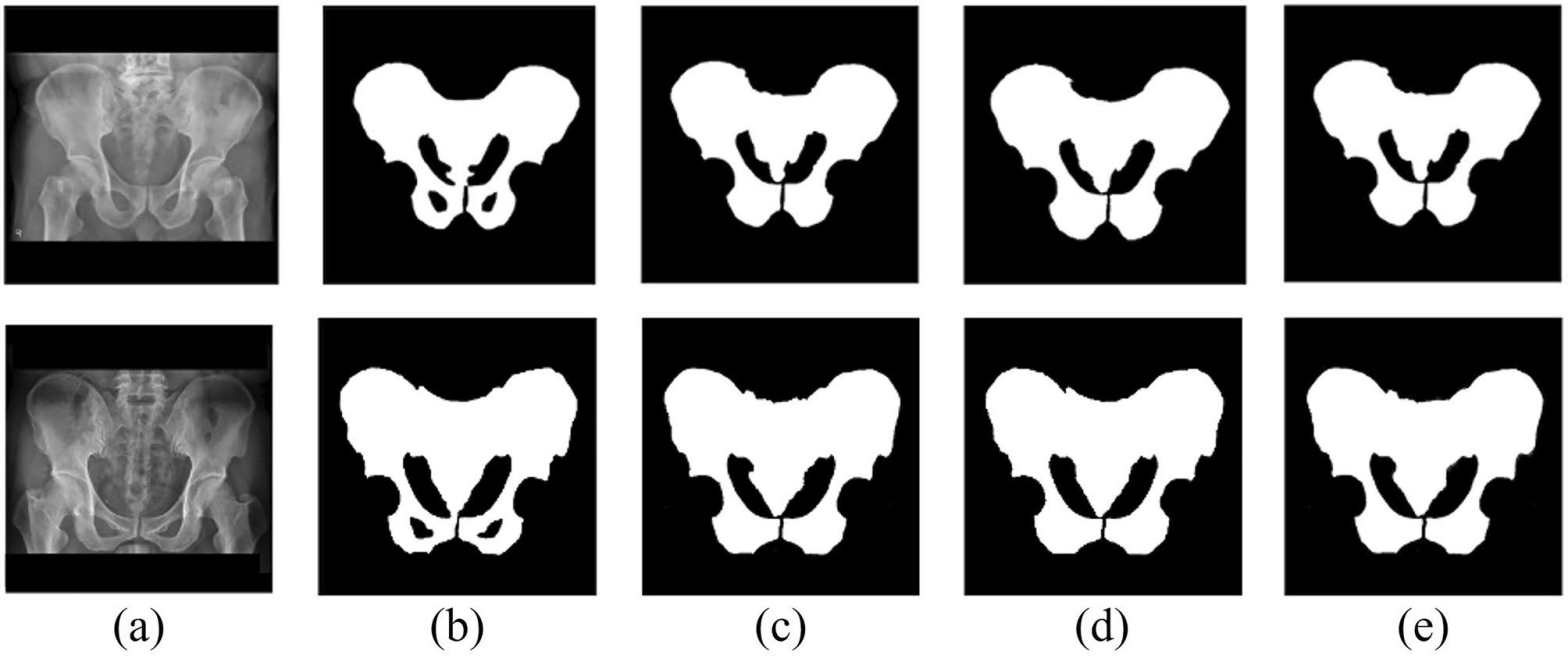
Comparison of pelvic segmentation based on Attention U-Net and Swin U-Net. (**a**) Original, (**b**) Gold standard, (**c**) Attention U-Net, (**d**) Swin U-Net, (**e**) U-Net.

In this study, we trained segmentation models using Attention U-Net, Swin U-Net, and U-Net models on pelvic X-ray images. Figure 5 shows a comparison between the pelvic regions segmented by trauma surgeons and those obtained using Attention U-Net, Swin U-Net, and U-Net models.

The results for ROIs are summarized in Table 1. Attention U-Net achieves an average sensitivity, specificity, and accuracy of 88.22%, 92.07%, and 90.80%, respectively. In contrast, Swin U-Net demonstrates an average sensitivity of 96.77%, a specificity of 98.50%, and accuracy of 98.03%, whereas U-Net achieves an average sensitivity of 88.40%, specificity of 91.24%, and accuracy of 84.46%. Table 2 shows the DSC changes associated with the three split models in the presence and absence of fractures. Following the analysis, Attention U-Net obtains a DSC of 83.66% for images with fractures and 83.78% for images without fractures. Swin U-Net achieves a DSC of 96.31% for images with fractures and 96.36% for images without fractures. U-Net achieves a DSC of 84.64% for images with fractures and 83.42% for images without fractures. The presence or absence of fractures does not result in a significant change in DSC.

**Table 1:** Performance comparison between Attention U-Net and Swin U-Net.

	U-Net	Attention U-Net	Swin U-Net
Sensitivity (%)	88.40 (± 3.91)	88.22 (± 3.60)	96.77 (± 0.14)
Specificity (%)	91.24 (± 1.74)	92.07 (± 2.27)	98.50 (± 0.08)
Accuracy (%)	91.05 (± 1.32)	90.80 (± 0.87)	98.03 (± 0.05)
DSC (%)	84.46 (± 0.66)	83.72 (± 0.89)	96.32 (± 0.13)

**Table 2:** Performance comparison between Attention U-Net and Swin U-Net.

	U-Net		Attention U-Net		Swin U-Net	
	Fracture	Non-fracture	Fracture	Non-fracture	Fracture	Non-fracture
DSC(%)	84.64	83.42	83.66	83.78	96.31	96.36

**Figure 6:**
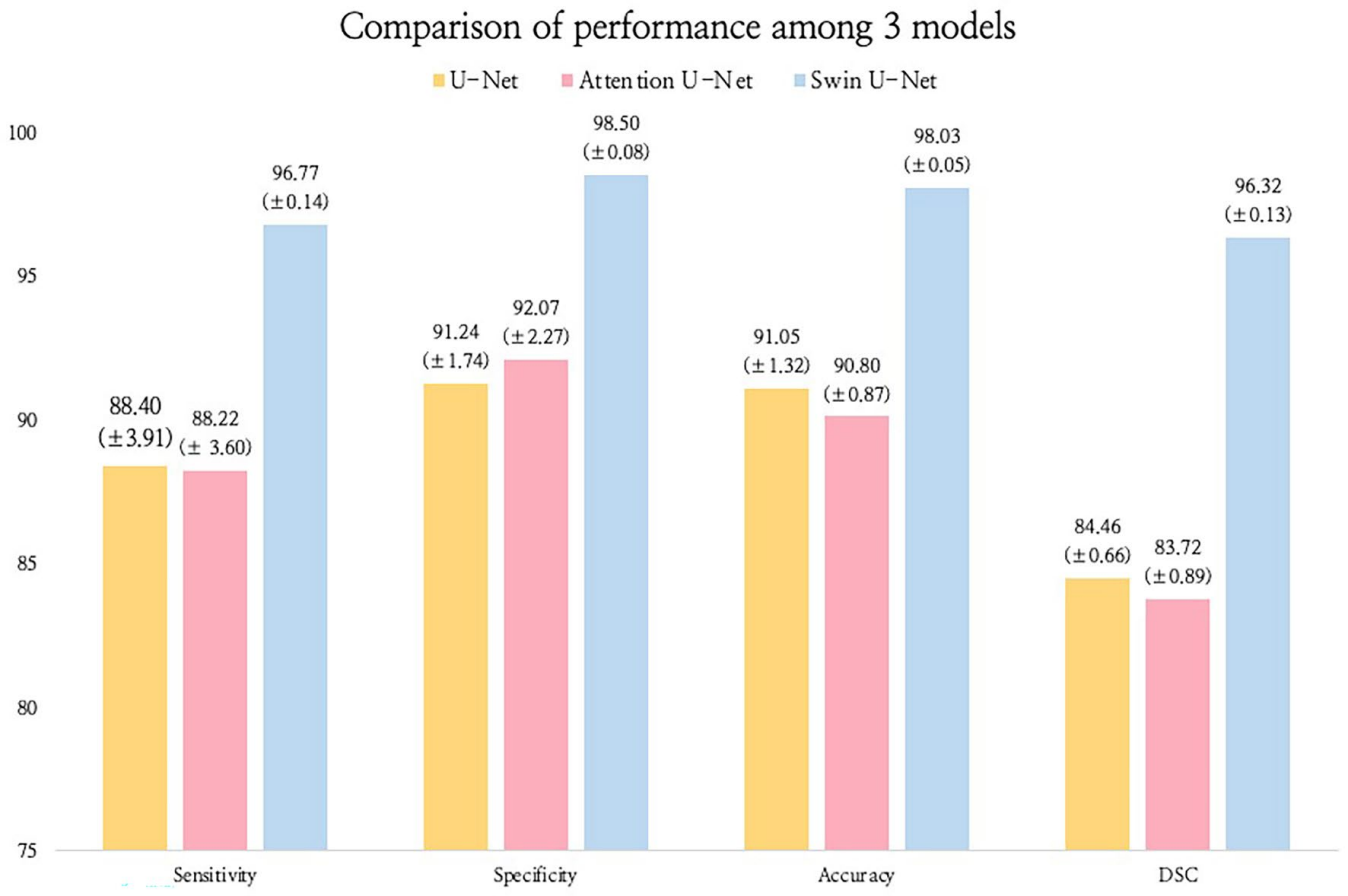
Performance comparison of the three models.

Figure 6 illustrates the performance differences among Attention U-Net, Swin U-Net, and U-Net. The Bland–Altman plots compare the test set prediction area of each model with the gold standard obtained from medical experts. The comparison results show that Swin U-Net outperforms the Attention U-Net and U-Net models in various evaluation metrics, with a significant difference observed for the DSC metric.

**Figure 7:**
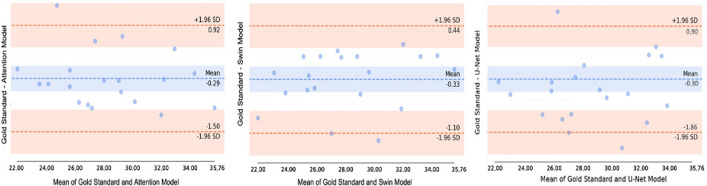
Bland–Altman plots comparing pelvic area from manual and deep-learning models.

The pelvic segmentation results between the ROIs obtained from Attention U-Net, Swin U-Net, and U-Net were further compared using Bland–Altman graphs (Fig. 7). The rationale behind selecting the Bland–Altman plot for comparing the Swin U-Net, U-Net, and Attention U-Net models lies in its ability to visually assess the agreement of the prediction results among the three models. This allowed us to determine the prediction consistency of the three models when applied to the same data. Additionally, examining the differences between the three models enabled us to understand how each model's predictions diverged, valuable for comparing the biases or consistencies in the predicted values between models. For differences in values clustered around the mean, indicating a strong agreement, the upper and lower limits of the Bland–Altman graph represent the standard deviation of the difference values, typically 1.96. This suggests that the differences were mostly within the range of 1.96 standard deviations. Hence, the differences between the ROI and segmentation results of the models were statistically significant, indicating the reliability of the model predictions.

**Figure 8:**
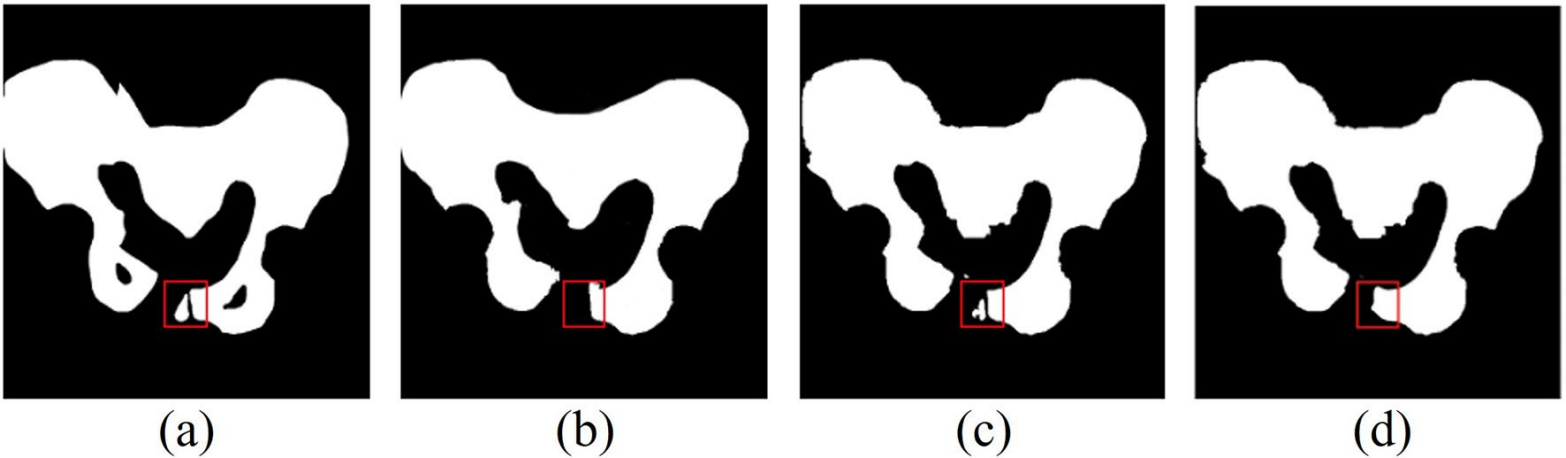
Comparison of fracture area within the segmentation results of Attention U-Net and Swin U-Net (**a**) Gold standard, (**b**) Attention U-Net, (**c**) Swin U-Net, and (**d**) U-Net.

Figure 8 shows the prediction results of each of the three segmentation models on an X-ray image with a fracture. The Swin U-Net demonstrates relatively greater accuracy in segmenting pelvic fracture images compared to Attention U-Net and U-Net. However, Swin U-Net has limitations in segmenting the left ilium, including the fractured area.

## Discussion

In this study, the trained models for pelvic region segmentation in X-ray images were evaluated and compared. Attention U-Net utilizes the attention technique to ignore or allocate less attention to areas unrelated to the gold standard, thereby reducing computational costs. In contrast, Swin U-Net learns by dividing the image into patches, facilitating a comprehensive understanding of the overall structure of the image and enabling identification of detailed features. Overall, both models used in the experiments exhibited excellent performance. From the experimental results, Swin U-Net was confirmed to exhibit higher sensitivity, specificity, accuracy, and DSC, compared to Attention U-Net. Regarding overall performance indicators, Swin U-Net demonstrated approximately 8% higher sensitivity, 6% higher specificity, and 8% higher accuracy than those of Attention U-Net. Among the performance indicators of the segmentation model, the most significant improvement was observed in DSC, which was approximately 13% higher for Swin U-Net. This suggested that Swin U-Net maintained stability and reliability across different input data compared to Attention U-Net, as its sensitivity did not change significantly with variations in input data. In terms of specificity, this implied that Swin U-Net outperformed Attention U-Net in accurately distinguishing the background of the image, excluding the pelvic bone region. Regarding accuracy, Swin U-Net achieved a higher accuracy rate than both Attention U-Net and U-Net in correctly classifying pelvic bone pixels in the image. Furthermore, in terms of DSC, the pelvic bone area predicted by Swin U-Net exhibited greater similarity to the actual pelvic bone area and was more accurate than those predicted by Attention U-Net and U-Net. In all experimental results, Swin U-Net outperformed Attention U-Net and U-Net. This superiority might be attributed to the efficiency of the Transformer series feature extraction function of Swin U-Net and reconstruction structure model. Swin U-Net simultaneously considered global and local features, allowing it to extract features that clearly segmented the detailed structure and boundaries of the pelvic region. In addition, extracting image features on a patch basis likely contributed significantly to segmenting the pelvic bone area using meaningful patch features. However, Attention U-Net tends to rely heavily on the efficiency of the attention mechanism. If the attention mechanism fails to extract appropriate features, the overall performance of the model may degrade. We speculate that the computational complexity of the attention mechanism might have led to overfitting during model learning and posed challenges in hyperparameter adjustment.

In a previous study, a DSC of 95.7% was achieved from CT images using a model with U-Net as the backbone of EfficientNet-B0^20^. Applying the Mask R-CNN model to X-ray images^21^ resulted in a DSC of 96%, whereas applying a 3D CNN model to multiparametric magnetic resonance imaging. mpMRI yielded a DSC of 85%^10^. In this study, the average DSC of 96.32% obtained using the Swin U-Net model indicated a performance improvement compared to previous studies.

However, the experimental results differed from those in the coccyx and sacral regions. This discrepancy may stem from the fact that the coccyx and sacrum have fused structures that differ from the ilium. The Bland–Altman graph shown in the experiment was used to compare the segmentation results of the three models. While both models exhibited cases exceeding the standard deviation, the distributions were relatively consistent. This implied that both models exhibited similar segmentation performance levels. However, the presence of outliers exceeding the standard deviation highlighted the need of improving the reliability and stability of model predictions. To achieve this, methods such as acquiring additional datasets and adjusting hyperparameters can be considered. In the experiments conducted in this study, Swin U-Net segmented the pelvic fracture images more accurately than Attention U-Net and U-Net. Given the complex shapes of pelvic fractures in images, accurate localization was crucial. Swin U-Net contributed to accurate localization by effectively segmenting the detailed structure and boundaries of the fracture site. However, it exhibited poor performance in segmenting the left ilium, including the fractured region, which could be attributed to the diversity of the images used for pelvic region segmentation training. The dataset included both normal images and images with pelvic fractures, leading to the pelvic region appearing fragmented rather than as a single cohesive structure. Including more pelvic fracture data in future studies could potentially enhance the segmentation performance of pelvic fracture images.

In conclusion, this study developed a deep learning model for pelvic region segmentation using X-ray images and compared it with other models. The results indicated that Swin U-Net achieved higher performance than Attention U-Net and U-Net. Further research based on these findings and subsequent improvements could potentially facilitate the application of this method in actual clinical practice.

## Data Availability

The data used to support the findings of this study are available upon request from the corresponding authors.
